# Making Art Therapy Virtual: Integrating Virtual Reality Into Art Therapy With Adolescents

**DOI:** 10.3389/fpsyg.2021.584943

**Published:** 2021-02-04

**Authors:** Liat Shamri Zeevi

**Affiliations:** Academic College of Society and the Arts, Netanya, Israel

**Keywords:** art therapy, virtual reality, adolescent, social difficulties, social anxiety

## Abstract

In recent years, the field of art therapy has sought to adapt traditional treatment approaches to today’s innovative technological environments when working with adolescent “digital natives.” In their clinic, art therapists often struggle with lack of cooperation when treating adolescents during sessions. This article presents two case studies that explore how Virtual Reality (VR) technology can be combined with traditional art therapy to treat adolescents suffering from anxiety and social difficulties. It is suggested that this type of technology may lead to a better understanding of the needs of adolescents by adopting their vantage point and hence better outcomes.

## Introduction

This article discusses an innovative way of integrating virtual reality (VR) into art therapy with adolescents and presents two case studies that illustrate this approach.

Art therapy is a form of dynamic emotional therapy where art materials, creative processes, and the creative product itself all serve as means of self-inquiry, self-expression, and a way to develop insights and create change (Malchiodi, 2011; Regev and Snir, 2017). VR is a technology that allows the individual to experience a reality different from the real world. It creates the illusion of another environment by generating digital, realistic or imaginary visual images (Biocca and Delaney, 1995). VR is a major component of computer games, films, educational materials and research (e.g., García-Betances et al., 2015; Freeman et al., 2017). Studies in the field of therapy and the implementation of VR as a therapeutic tool in mental health are also on the rise (Riva et al., 2019). The most common form of VR in the mental health field is known as exposure therapy (VRE) where VR replaces the sequential progression of physical exposure which allows the client to have step by step control over the experience. For example, VRE is used to treat phobias (Botella et al., 2017), trauma and post-trauma (Gonçalves et al., 2012), body image and eating disorders (Clus et al., 2018), as well as anxiety disorders, including social anxiety (Anderson et al., 2013; Shalom et al., 2015; Dechant et al., 2017(. Social anxiety disorder (SAD) is defined as a marked and persistent fear of social or performance situations in which embarrassment may occur, resulting in significant distress and difficulties in functioning (American Psychiatric Association, 2013). SAD is the third most common mental health disorder after depression and substance abuse, with lifetime prevalence rates of around 12%. It emerges in adolescence, with 90% of all cases occurring before the age of 23 with a median age at onset of 13 (Kessler et al., 2005).

In the field of art therapy recent research has focused on adapting and connecting the technological environment and the therapeutic environment for general use and specifically for adolescents (e.g., Darewych et al., 2015; Garner, 2017; Hacmun et al., 2018; King and Kaimal, 2019; Kaimal et al., 2020). Ohrius and Malchiodi (2018) examined the interactive and sensory aspects of digital media and suggested that there are clear-cut differences between the sensory experiences provided by digital technology and more traditional creative materials, thus making digital media a viable alternative that can contribute to self-expression and therapeutic change.

Art therapy in VR can be viewed as a kind of collage, in which images or selected parts of images are used, cut and attached to a new work that expresses a different content, thus allowing for the expression and reconnection of different components of the human psyche (Hinz, 2019). In this way the product can act as a container for typical adolescent feelings, such as anger, guilt, and confusion, and enable common defense mechanisms such as disengagement and dissociation to be expressed (Shwartz, 2000).

Studies have argued that daily life of today’s children and adolescents operates simultaneously in two primary spaces: the interpersonal everyday space and the digital space (Chandra, 2016). Adolescents are often referred to as “digital natives” (Prensky, 2001) because they were born in the digital age and consider technology to be a native language. Adolescents often perceive the use of technology in therapy as a pleasant and comfortable extension of a familiar form of discourse. A survey (Autry and Berge, 2011) showed that younger individuals were more likely to incorporate technology into their learning environment. It is not uncommon in clinical settings to encounter adolescents who prefer to share their feelings via a picture, text, or video rather than holding a conversation or making a free drawing. In clinical work, it is often challenging to work with adolescents who do not cooperate or comply (as part of the natural rebellion of adolescence) in the traditional sense during art therapy sessions. The therapeutic approach in these case studies assumes that the encounter between the world of art and the world of VR creates a rich triangular relationship between the therapist, the adolescent, and the virtual artwork. The presence of traditional art materials and virtual art material in the therapy room provides clients with the opportunity to take part in a visual creative experience that utilizes their imagination and enables the symbolic and non-verbal expression of unconscious content (Schaverien, 2000; Case and Dalley, 2014).

The case studies below describe an attempt to implement VR with traditional art therapy treatment for adolescents experiencing anxiety and social difficulties.

## The Context: Integrating Virtual Reality Into Art Therapy and Its Components

### VR in Therapy

**FIGURE 1:**
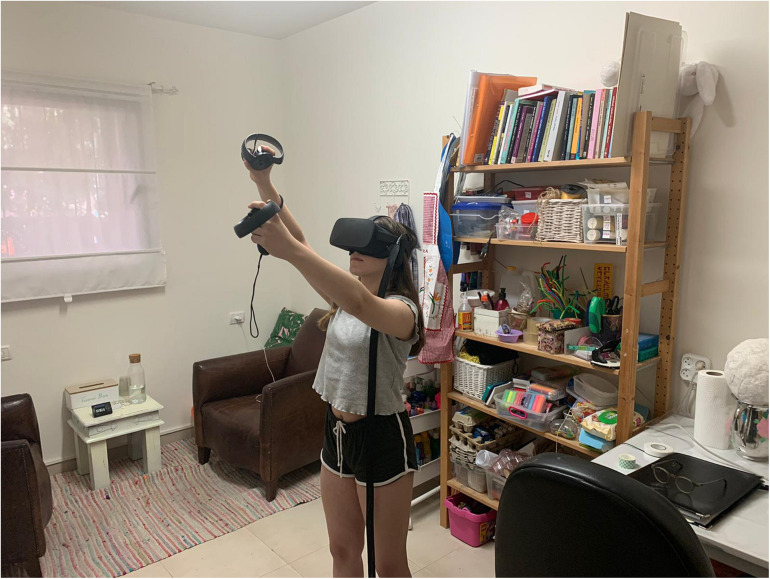
The setting and therapeutic space are influenced by the VR’s creative environment.

As shown in Figure 1, the setting and therapeutic space are influenced by the VR’s creative environment, which is driven by the nature of the technology. When using a VR device (in this case an Oculus Rift device), participants wear a Head Mounted Display (HMD) and use Tilt Brush software by Google^1^ within a closed virtual space. Participants operate two hand-held controllers which allow for Motion Capture and Creation (MOCAP)^2^. One controller is designed for drawing and the other is for the controls and color menus simulating classic painting with a color palette and paintbrush. Both hand and full body movements are involved. Free movement in space is made possible through HMD-lit grids that mark the boundaries of the physical space. In this way the client can physically and metaphorically gain control and define the boundaries of the treatment room (it should be noted that the anatomical structure of the controllers look similar to that of the Sony PlayStation/X-box console and therefore are usually intuitive for teens and do not require a lengthy period of adaptation).

Correcting mistakes is easy and instantaneous and simply involves deleting adding and modifying elements. The equipment is relatively simple and its use does not require organization or a special setup in the clinic. The preservation and printing of the works is simple and no physical space for storage is required (Choe, 2014). Note that wearing the HMD means that there is no eye contact between the therapist and the client so that communication can only be verbal. The therapist can only view the client’s ongoing artwork on a PC/television screen, which provides a limited 2D version of the 3D artwork.

**FIGURE 2:**
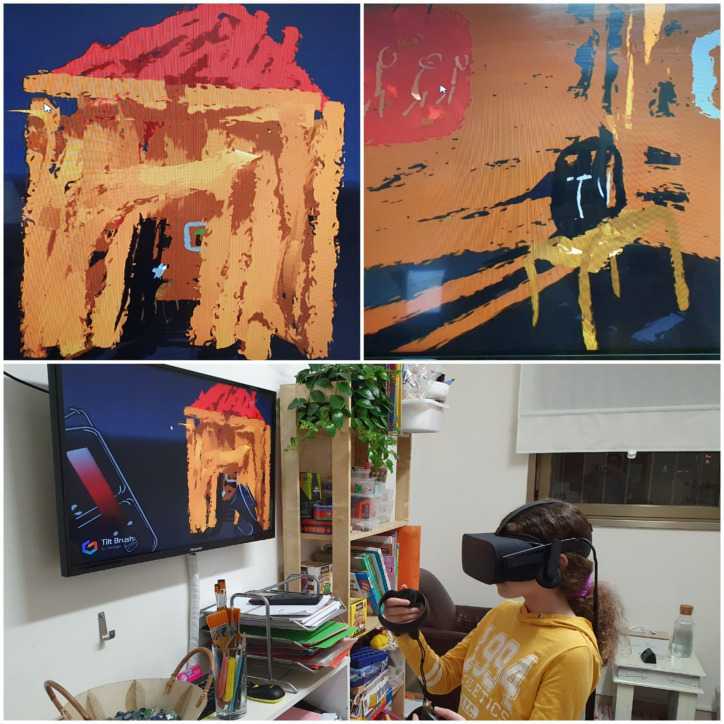
House via multiple angles.

Drawing in VR involves combining elements digitally from the world of painting and sculpture that are used traditionally in art therapy sessions, such as line, form, marks, and color. However, VR outputs are distinctive in a number of ways. The artwork itself is virtual and therefore lacks concrete physicality and tactile feedback. The drawing is executed in 3D and allows the viewer to observe the work from multiple angles, including from inside the work itself as shown in Figure 2 (entering a house via multiple angles).

The virtual space also lets art makers use material that has unworldly properties since the work is not subject to the laws of physics and the material and the space can include fantasy motion and dynamic changes in limitless space. However, when difficult emotional content is expressed, the art therapist can still accompany the client, by maintaining verbal communication even though the client is still wearing the VR glasses and is still immersed in the experience. Alternatively, the client can control the situation by removing the VR glasses, and return to the secure, familiar environment in the physical space of the clinic. This can then allow for the physical processing of traditional art materials (such as various kinds of paints including oil, gouache, acrylic, crayons, markers, pencils, clay, paper mache, wool, fabrics, glitter, wire, plaster, pipe cleaners, wooden skewers, and others) in relation to the virtual experience. Finally, in VR work, the vantage point for viewing can be controlled with a first-person perspective (1PP) or “through the eyes of the other,” a third person perspective (3PP).

## Programmatic Case Studies: Integrating Virtual Reality Into Art Therapy

### Participants

The case studies below describe VR art therapy treatments for a boy, aged 16, and a girl, aged 13, suffering from anxiety and social difficulties. These were taken from the case log of therapists working in a private art therapy clinic in Israel. Participants signed consent forms authorizing the publication of their works.

### General Procedure

Each VR art therapy session is made up of three main segments. At the beginning of the session, the therapist discusses which media the client wants to use. If the client prefers to use art materials, s/he will work using the traditional work station (table, chair, and art materials). If the client chooses to use VR, the therapist will make sure that the HMD is appropriate and the right size for the client’s head measurements and that s/he remembers how to use and draw with the Google Tilt Brush software (providing familiarization with the physical space), demonstrates the layout of the virtual controllers to the client (two handheld controllers) and sets the Tilt Brush software on a clean 3-D canvas.

During the creative process, the therapist accompanies the client in the virtual space on a 2D monitor that displays what the client is creating in his/her 3D world, as well as by talking to the client (note that the client cannot see the therapist and can only hear him/her when wearing the HMD). Occasionally there will be alternation between the traditional and digital media when for example the client asks to scan an image or picture into the digital media so that he/she can relate to it while creating the virtual work. Alternatively, printing the digital work can also be done to combine the VR output with traditional art materials. At the end of the session, the client removes the HMD and the therapist mediates the content that emerged “then and there” (in the virtual media) and in the “here and now.” This is a significant part of the therapeutic process that generates and preserves the integration of the various parts of the self during creative work with VR. It serves as a processing tool to clarify issues, such as “What did I create?” “What allowed me to create and what prevented me from creating?” “What were my conflicts, feelings, and emotions during the creative process?” The entry into and departure from the virtual space does not allow an escape into this space, but rather promotes deep emotional work with the client’s internal content.

### Ethical Aspects

The adolescents and their parents gave their full consent for the publication of the case study and for the reproduction of the creative material documented in this article. The case studies are written so that both the rights and the privacy of the participants are protected.

The art therapy sessions took place in private clinic and adhered to all the guidelines for responsible therapeutic practice as stipulated in the American Art Therapy Association (2013) guidelines for ethical principles.

The two case studies below illustrate the advantages and disadvantages of integrating virtual art-making into art therapy for adolescents experiencing anxiety and social difficulties.

### Case Study 1: Eric

Eric (pseudonym), a 16-year-old boy, is a curious, short, introverted, and inhibited adolescent. Eric’s treatment lasted 8 months in total. He was oriented to art therapy because of his difficulties in forming social relationships, experiences of anxiety, low self-esteem, and academic difficulties at school. In the first few years of school, he integrated well but exhibited hypersensitivity and had outbursts and manifestations of anger when he did not get his way. In his free time, Eric enjoys playing computer games and watching television.

He has an active internet persona and a popular YouTube channel that gets many “Likes” and provides him with a platform to form meaningful connections with the world and his peers. Eric believes that his internet persona is the one that truly represents him, and that he embodies this side of himself to a lesser extent in real life. For many years Eric avoided therapy of any kind (he had tried different types of treatment, but would not cooperate with his therapists). At the beginning of the therapy, his parents reported that he was opposed to attending the sessions and that they practically had to “force” him to go. However, during the sessions, this form of resistance was not felt and Eric cooperated well. The therapeutic goal was to help Eric lower his anxiety level, raise his self-confidence, and form meaningful social bonds.

**FIGURE 3:**
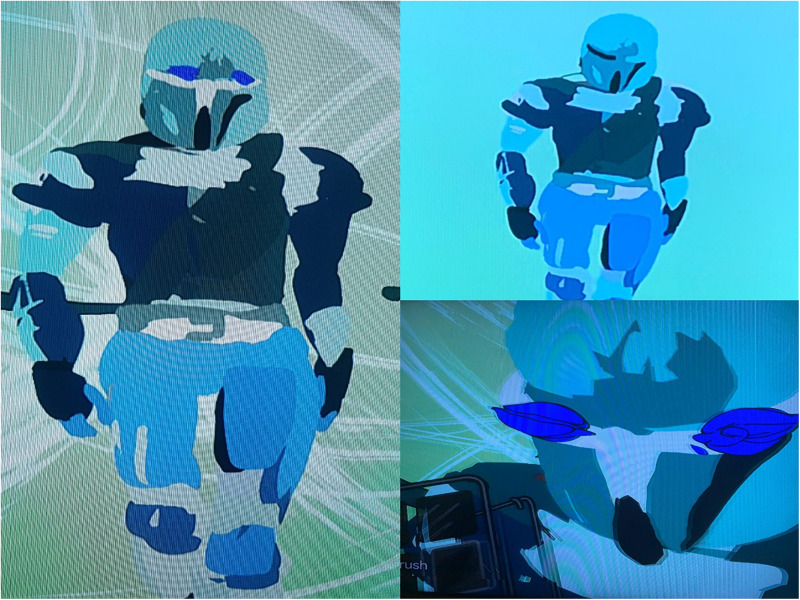
Eric’s Iron man.

In the first four sessions, Eric was proficient in creative work in VR and it was clear that he was familiar with the technology. This seemed to make it easier for him to make initial contact and continue to receive treatment (although according to his parents he still did not want treatment at this point). After the first session where I explained the therapeutic work in VR to Eric, he would enter the room with a humiliated look, put on the HMD and create for the next 45 min in VR (with short breaks). He would sometimes tell me what he had created but mostly worked silently and I watched his work from my screen. I felt a bit secluded and sometimes almost like I peeked at his work, since we do not see each other and do not make eye contact. In these sessions, he created aggressive content relating to “good vs. bad” and “small vs. large,” where most of the small evil forces overcame the larger benevolent forces. In the fifth session, he worked as usual throughout the session, but began to talk and describe what was happening to the characters. During the creative process, Eric explained with his face covered by the HMD that: “This is Iron Man, he is very strong, he wears a silver mask and has blue eyes, and you can see his eyes through the mask.” In this session, Eric insisted (as usual) on working creatively until the end of the session and refused to talk at the end when his face was visible and we had eye contact. At the next session, Eric brought a small mask with him. He sat down in front of me for the first time, although his gaze still lowered, and said that he was given the mask by his parents when they had toured Venice a few years earlier. He was much younger when they were away and he missed them very much, but he was afraid to be treated “like a baby” if he expressed his longing for them, so he remained silent and played on the computer in his room. He mentioned that since then he has been wearing a kind of mask, like a wall between himself and his surroundings. Eric asked me to scan the mask into the digital software. He began by reducing and enlarging the mask and then incorporating it into his work. He then explained: “The mask isn’t actually made of iron (the iron man he created), but we call it that. He is an ordinary man, but he has powers to turn his body into iron. If he is in the middle of his work and something happens, he turns himself into the Iron Man.” Eric continued to create in VR in the next few sessions when he was already able to take off the HMD (mask), talk to me directly, and even look straight at me from time to time. He described it as follows: “He is a man with many strengths, but his blue eyes are only for appearance.” After a few more sessions when Eric felt comfortable in the clinic, he noticed a white paper mask in the clinic. He placed the mask over his face and asked if he could color it. He started working with watercolors and created a “colorful soft mask” for himself. At the same time, Eric casually mentioned that he had connected with a friend he likes from YouTube on his Instagram account and suggested meeting him face-to-face. This was the start of Eric’s process of gradually removing his “iron mask” and communicating with others in a personal way and not only via his internet persona. As Eric transitioned during the sessions between the VR and the traditional art therapy spaces by making connections between emotions and content he also began to make the connections between his Internet persona and the real world (Figure 3). The treatment ended after 8 months, when Eric felt that his anxiety level had dropped and that he was able to form a number of social connections. It should be noted that at the same time he did not part from his internet persona and continued to cultivate it as well.

This case study suggests that it is important for these adolescents to recognize their parallel existence (in reality and in the online world), and to consider where they are personally and emotionally positioned and where their energy is invested. In this context, VR played an intermediary role by allowing Eric to take a break from reality and be present with his online strengths and traits. The therapist acknowledged that these were dominant important spaces and did not reject or disregard them. Many aspects of VR art and children’s play can be understood through the concept of creativity as defined by Winnicott (1971). Play and creative work in VR appear to be embodiments of symbols in that they use objects to represent perceptions and emotions, and bridge between internal and external worlds. In this context, the role of the therapist is to lead the client from a state of inability to play to a space of enabled play, particularly for individuals who often find it difficult to bridge the gap between the concrete world and the symbolic and emotional world. This “intermediate space” may also allow for therapeutic work on clients’ difficult and inaccessible emotional content that accelerates the therapeutic process. In this way, the VR creative work expands clients’ modes of expression and communication, enabling them to express their inner world and emotions non-verbally. Landy (1994) coined the term “aesthetic distance” to describe the activation of therapeutic processes where the client has no fear of being unmoored as a result of the strength of the emotion or from losing the ability to respond emotionally. This borderline condition allows for contact and connection with the point of crisis. In crisis and trauma situations, the aesthetic distance helps clients deal with difficult emotions associated with the event and address them. In Eric’s case, the aesthetic distance when using VR allowed him to contact and connect with his feelings.

In the therapeutic context, the aesthetic distance relates to the degree of proximity of the client with his/her feelings, thoughts and perception of the self. When too close to the experience, the self can get lost in it; when too distant the risk is to become disconnected. This points to the importance of finding the right distance or the perfect balance. In particular, entering too rapidly into this space (which is enabled by VR), can touch on and reveal deep emotional content, can threaten the client and sabotage and impair the therapeutic process if a personalized approach is not taken. On a concrete level, adolescents are often surprised when they encounter a computer savvy therapist who can “speak their language.”

Another way of looking at the essence of this encounter is to compare VR to collage. The versatility and flexibility of collage offer endless possibilities for organizing the components, placing them next to each other in unconventional ways corresponding to a transformative experience with images and meanings (Johnston, 2007; McClellan, 2011). Working with virtual material seems to have allowed Eric to experience the disassembly and assembly process, both concretely and symbolically. This process took place during his creative work, but this time arose from a place of control and choice, thus providing Eric with a space in which he could observe the experience of disintegration or splitting (between his concrete and virtual character, between the persona and the mask) and not just experience it. Hence, this work while engaging in the selection and organization of virtual materials, can be symbolic of creating order from the fragmentary aspects of life, as well as from chaotic emotions (Malchiodi, 2002; Yedidiaand Levine-Keini, 2005).

**FIGURE 4:**
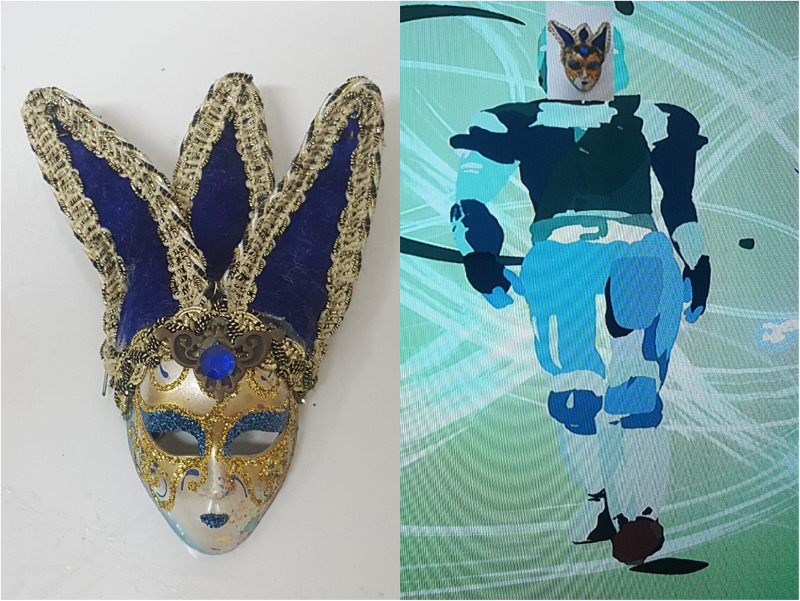
Eric’s mask.

Another technique made possible through VR technology is the ability to upload images and photographs in 2D, as well as 3D objects, artworks and various figures into the virtual space. These can be viewed from all sides in 360 degrees thus creating a new reality in which the client is an integral part of the story and not just an observer, as Eric did with the mask (Figure 4). Thus, the VR user experiences a phenomenon which is described as an “immersive experience” that stimulates many of the senses ensuring the user feels like they really are in that virtual environment. This is not part of the therapist’s experience who can only monitor the results in 2D on the screen. This context sheds light on the mask that Eric brought to the treatment room. The purpose of a mask in art therapy is to help individuals understand themselves and their relationship with the Other (e.g., Baptiste, 1989; Meiri, 2013). Keats (2003) suggested that the use of the self-masking technique may heighten and strengthen the development of the therapeutic alliance, in that the mask is used as a non-verbal mode of communication which can enable the establishment of verbal communication. In addition, self-masks allow clients to address issues that arise during treatment, since the mask allows for the establishment of the roles of identification, concealment, nourishment and/or change. Siano (2016) suggested that found objects are in fact the same objects people use to invent and mediate personal narratives. Without words, a connection is made between an individual and a meaningful object. The ability to transfer objects and/or figures into the VR world enables a new creative and playful space. By choosing the object and engaging with it in the 3D space, the client can tell his/her story without words while remaining within the projective imagery world. The use of objects in the VR world gives the client freedom of choice to select a medium and an image which can symbolize first step in “re-writing” a life story that the therapist can help charge with meaning.

### Case Study 2: Alma

**FIGURE 5:**
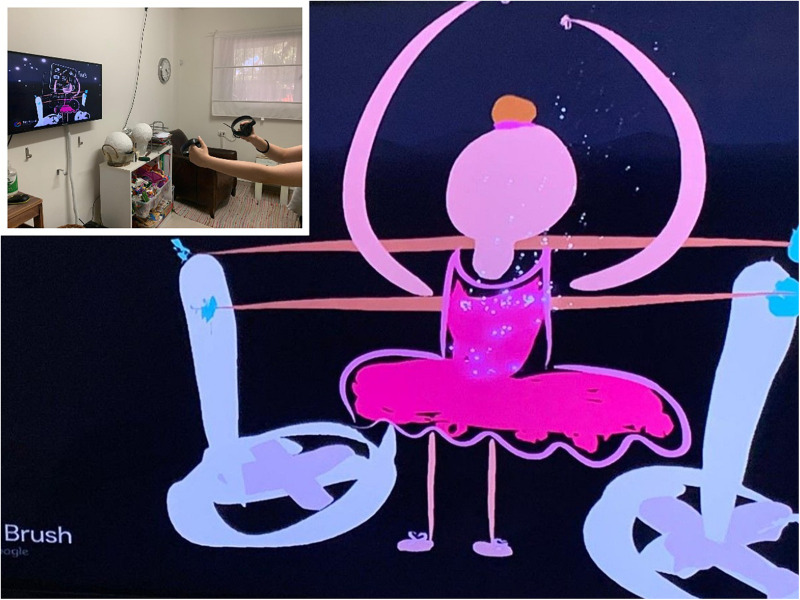
Alma’s dancer.

Alma (pseudonym), a 13-year-old girl, is the daughter of a single mother. Alma’s treatment lasted 6 months. The therapeutic goal was to improve her self-body image and help her form social bonds with peers. Alma is clever, shy, and a perfectionist who started therapy as a result of her social difficulties and poor body image. Alma dances for many hours after school and she has a very rigid perception of the world. When her peers reach out to her, she experiences it as an attack on her private space. She feels distressed at school every day and has a strong sense of loneliness and alienation from others. Alma reports she feels fat (although she is slender), she usually wears baggy clothing and walks slightly hunched over. When she came to therapy and put on the HMD for the first time, her whole attitude changed. Suddenly she moved lightly and gracefully in the room, her head was upright and it looked like she was dancing while engaging with the media. In the first session, she had a difficult time getting used to the controls and became upset and frustrated. She frequently removed the device from her head, only to put it back on. Alma found it difficult to contact me directly at this time. This behavior raised a number of doubts about working with her in this medium and after the first meeting I was skeptical about the advantages of the combined treatment in VR with her. In the second session, she managed to operate the system much better, and when the VR experience was over, she commented that she felt “successful” and then added: “I feel that I have achieved something.” This suggests that work with the VR technology requires a learning curve, in that Alma needed time to get familiar with the system functionalities. In the third session, Alma again experienced frustration while trying to draw a dancing figure. She wanted to make one of the images smaller but failed. She commented: “I feel like at school when I want to be closer to the other children and feel they don’t want to get close to me” (her lack of eye contact with me may have made it easier for her to discuss this spontaneously), “I’m angry and frustrated, I feel rejected, I feel that they don’t like me.” She suddenly seemed shocked: “Oh, I can’t believe I said that, I have never said that to anyone.” I reflected on her difficulty, her courage, her ability to say things and I asked her if she often felt that way. She replied: “Yes, when I want to do something in dancing and I can’t do it. In dance class, I’m really good, but the teacher doesn’t like me. She gives the lead roles to another girl and we have to dance around her. Like I’m dancing right now and trying to get closer to the dancer. I cannot do it; I would also like to be the soloist. It really frustrates and annoys me.” Later in the process, when she accomplished something, she felt a great experience of success. In the next few sessions, Alma continued to create her dancer. When she looked at the dancer, she talked about the problems she has with her body image and how challenging it is to wear a leotard every day and feel that her body is under scrutiny. Gradually, I noticed that Alma wore more close-fitting clothing that complimented her slim build and she became fond of and identified with the slender dancer she created, although at first, she struggled to connect with it and even experienced slight jealousy. Toward the end of the treatment she reported that she started approaching her classmates and was less concerned about their criticism and understands how difficult it was for them to approach her in the past (Figure 5).

Several studies (e.g., Banakou et al., 2013) have shown that changing the visual perspective in VR from the personal perspective to the perspective of the other may result in a significant change in the perception of social behavior that can lead to changes in the experience (for example, by experiencing what an obese child experiences, the therapist may be more empathetic to his/her worldview and difficulties in the future) and in cognitive processing. These changes have significant therapeutic implications; for example, in relation to the experience of control, since images can be easily modified and preserved while experimenting with other qualities (Libby et al., 2009). Several art therapists have expressed their concerns that technology is causing alienation and dehumanization and impairing the imagination (Carlton, 2014). However, Orr (2011) argued that the art therapist should relate to the digital media as a mediator, much like a brush in the hand of a painter. The existence and quality of the relationship between the therapist and client depends on their interpersonal interactions and not on the choice of mediator.

Thus, through visual symbolization negative emotions appear to be expressed through virtual materials and physical actions, without having to direct them toward another person. Later on, the client can go back to look at the visual products and to see whether there has been a change in the visual symbolization. Thus, the content takes on a tangible presence that can be reflected, and the very personal becomes visible. In addition, the ability to examine and observe the products of virtual art from a safe psychological distance make it possible to reprocess images and in so doing acquire a form of soothing control that can reduce anxiety (Anderson, 1995).

Another equally important issue is the ability to assimilate within the virtual environment, which is characterized by high immersive qualities that enable 360° visual perspectives (Slater et al., 2008). The sense of presence can be a powerful therapeutic tool for promoting change during the process of self-reflection because it gives the user the opportunity to “experience” him/herself differently (Riva et al., 2016), much like Alma experienced her dancer in her therapy sessions.

## General Discussion

Thus overall, the VR technique may be particularly beneficial in treating adolescents who find it difficult to create traditionally in the art therapy clinic. VR can also be a therapeutic alternative for clients who are afraid of making mistakes in their work because it allows for an experiential exploration without any physical or real-world implications. For clients in VR art therapy who do not perceive themselves as imaginative individuals, the artwork in this setting may help them develop concrete ideas and find ways to express themselves that are not available through other guided imagery techniques.

### Limitations and Future Work

When the art therapist is watching the client work on the monitor and not viewing the actual client, a disconnection from the client may occur as a result of the split in attention. In addition, users (mainly in the first sessions) may experience slight dizziness, a feeling that in extreme cases can last for a short time after the therapy session comes to an end. This makes it crucial for future studies to explore the use of VR in art therapy in relation to clients with different clinical, physical, or mental states. Follow-up studies can be conducted to assess the impact of VR in relation to art therapy for different treatment groups (children, adolescents, adults, mental health, etc.) in the short and long term. Finally, clinical treatment and quantitative research on anxiety reduction should be pursued to better understand which therapeutic needs are addressed and fulfilled by integrating virtual media into the traditional arts therapy toolbox.

## Data Availability Statement

All datasets generated for this study are included in the article/supplementary material, further inquiries can be directed to the corresponding author/s.

## Ethics Statement

Written informed consent was obtained from the individual(s) for the publication of any potentially identifiable images or data included in this article.

## Author Contributions

The author confirms being the sole contributor of this work and has approved it for publication.

## Conflict of Interest

The author declares that the research was conducted in the absence of any commercial or financial relationships that could be construed as a potential conflict of interest.

## Footnotes

1 www.TiltBrush.com

2 The Google Tilt Brush software requires a computer with similar (or higher) capabilities: (a) A computer processor: Intel Core i5-8400 CPU 2.8 GHz; (b) A graphics card: NVIDIA GeForce GTX 1060 6 GB; (c) Internal memory: 8 GB RAM; (d) Output: 1 HDMI, 3 USB 3.0; and (e) An operating system: Windows 10 64 bit.

## References

[B1] American Art Therapy Association (2013). *Ethical Principles for Art Therapists.* Mundelein, IL: American Art Therapy Association

[B2] American Psychiatric Association (2013). *Diagnostic and Statistical Manual of Mental Disorders*, 5th Edn, Washington, DC: American Psychiatric Association.

[B3] Anderson F. E. (1995). Catharsis and empowerment through group claywork with incest survivors. *Arts Psychother.* 22 413–427. 10.1016/0197-4556(94)00046-8

[B4] Anderson P. L. Price M. Edwards S. M. Obasaju M. A. Schmertz S. K. Zimand E. (2013). Virtual reality exposure therapy for social anxiety disorder: a randomized controlled trial. *J. Consult. Clin. Psychol.* 81:751. 10.1037/a0033559 23796315

[B5] Autry A. Berge Z. (2011). Digital natives and digital immigrants: getting to know each other. *Industr. Commercial Train.* 43 460–466. 10.1108/00197851111171890

[B6] Banakou D. Groten R. Slater M. (2013). Illusory ownership of a virtual child body causes overestimation of object sizes and implicit attitude changes. *Proc. Natl. Acad. Sci. U.S.A.* 110 12846–112851. 10.1073/pnas.1306779110 23858436 PMC3732927

[B7] Baptiste D. A. (1989). Using masks as therapeutic aids in family therapy. *J. Family Ther.* 2 45–58. 10.1046/j.1989.00332.x

[B8] Biocca F. Delaney B. (1995). “Immersive virtual reality technology,” in *Communication in the Age of Virtual Reality*, Vol. 15 eds Biocca F. Levy M. R. (Hillsdale, NJ: Erlbaum), 57–421.

[B9] Botella C. Fernández-Álvarez J. Guillén V. García-Palacios A. Baños R. (2017). Recent progress in virtual reality exposure therapy for phobias: a systematic review. *Curr. Psychiatry Rep.* 19:42. 10.1007/s11920-017-0788-4 28540594

[B10] Carlton N. R. (2014). Digital culture and art therapy. *Arts Psychother.* 41 41–45. 10.1016/j.aip.2013.11.006

[B11] Case C. Dalley T. (2014). *The Handbook of Art Therapy.* Abingdon: Routledge.

[B12] Chandra A. (2016). Social networking sites and digital identity: the utility of provider-adolescent communication. *The Brown University Child and Adolesc. Behav. Lett.* 32 1–7. 10.1002/cbl.30107

[B13] Choe S. (2014). An exploration of the qualities and features of art apps for art therapy. *Arts Psychother.* 41 145–154. 10.1016/j.aip.2014.01.002

[B14] Clus D. Larsen M. E. Lemey C. Berrouiguet S. (2018). The use of virtual reality in patients with eating disorders: systematic review. *J. Med. Internet Res.* 20:e157. 10.2196/jmir.7898 29703715 PMC5948410

[B15] Darewych O. H. Carlton N. R. Farrugie K. W. (2015). Digital technology use in art therapy with adults with developmental disabilities. *J. Dev. Disabil.* 21 95–102.

[B16] Dechant M. Trimpl S. Wolff C. Mühlberger A. Shiban Y. (2017). Potential of virtual reality as a diagnostic tool for social anxiety: a pilot study. *Comput. Hum. Behav.* 76 128–134. 10.1016/j.chb.2017.07.005

[B17] Freeman D. Reeve S. Robinson A. Ehlers A. Clark D. Spanlang B. (2017). Virtual reality in the assessment, understanding, and treatment of mental health disorders. *Psychol. Med.* 47 2393–2400. 10.1017/S003329171700040X 28325167 PMC5964457

[B18] García-Betances R. I. Arredondo Waldmeyer M. T. Fico G. Cabrera-Umpiérrez M. F. (2015). A succinct overview of virtual reality technology use in Alzheimer’s disease. *Front. Aging Neurosci.* 7:80. 10.3389/fnagi.2015.00080 26029101 PMC4428215

[B19] Garner R. L. (ed) (2017). *Digital art Therapy: Material, Methods, and Applications.* London: Jessica Kingsley Publishers.

[B20] Gonçalves R. Pedrozo A. L. Coutinho E. S. F. Figueira I. Ventura P. (2012). Efficacy of virtual reality exposure therapy in the treatment of PTSD: a systematic review. *PLoS One* 7:e48469. 10.1371/journal.pone.0048469 23300515 PMC3531396

[B21] Hacmun I. Regev D. Salomon R. (2018). The principles of art therapy in virtual reality. *Front. Psychol.* 9:2082. 10.3389/fpsyg.2018.02082 30429813 PMC6220080

[B22] Hinz L. D. (2019). *Expressive Therapies Continuum: A Framework for Using Art in Therapy.* Abingdon: Routledge.

[B23] Johnston K. A. (2007). *A Phenomenological Study Exploring the Cognitive and Emotional Experience of Using Magazine Photo Collage Media in Art Therapy*. unpublished master’s thesis, Drexel University, Philadelphia, PA.

[B24] Kaimal G. Carroll-Haskins K. Berberian M. Dougherty A. Carlton N. Ramakrishnan A. (2020). Virtual reality in art therapy: a pilot qualitative study of the novel medium and implications for practice. *Art Ther.* 37 16–24. 10.1080/07421656.2019.1659662

[B25] Keats P.A. (2003). Constructing masks of the self in therapy. *Constructivism Hum. Sci. ProQuest Psychol. J.* 8 105–123.

[B26] Kessler R. C. Berglund P. Demler O. Jin R. Merikangas K. R. Walters E. E. (2005). Lifetime prevalence and age-of-onset distributions of DSM-IV disorders in the National Comorbidity Survey Replication. *Arch. Gen. Psychiatry* 62 593–602. 10.1001/archpsyc.62.6.593 15939837

[B27] King J. L. Kaimal G. (2019). Approaches to research in art therapy using imaging technologies. *Front. Hum. Neurosci.* 13:159. 10.3389/fnhum.2019.00159 31156413 PMC6534043

[B28] Landy R. J. (1994). *Drama Therapy: Concepts, theories and Practices.* Springfield, IL: Charles C Thomas Publisher.

[B29] Libby L. K. Shaeffer E. M. Eibach R. P. (2009). Seeing meaning in action: a bidirectional link between visual perspective and action identification level. *J. Exp. Psychol. Gen.* 138 503–516. 10.1037/a0016795 19883133

[B30] Malchiodi C. A. (ed) (2011). *Handbook of Art Therapy.* New York, NY: Guilford Press.

[B31] Malchiodi C. A. (2002). *The Soul’s Palette: Drawing on Art’s Transformative Powers.* Berkeley, CA: Shambhala Publications.

[B32] McClellan J. L. (2011). *The Use of Collage in Leadership Education. Leadership Advance Online, XXI.* Available online at: https://kevek.co/16.pdf (accessed October 16, 2020).

[B33] Meiri K. (2013). The Mask as an Artistic Tool of Exploration: Unmasking the *‘True Self’* and the *‘False Self’.* Thesis submitted in partial fulfillment of the requirements for Master’s degree, University of Haifa, Hebrew.

[B34] Ohrius J. Malchiodi C. (2018). “Virtual reality art therapy,” in *Handbook of Art Therapy and Digital Technology*, ed Malchiodi C. (London: Jessica Kingsley Publishers), 215–229.

[B35] Orr P. (2011). “Social remixing: art therapy media in the digital age,” in *Materials & Media in Art Therapy*. ed Moon C. H. (Abingdon: Routledge), 121–132.

[B36] Prensky M. (2001). Digital natives, digital immigrants. *Horizon* 95, 1–6.

[B37] Regev D. Snir S. (2017). *Parent-Child Art Psychotherapy.* Abingdon: Routledge.

[B38] Riva G. Baños R. M. Botella C. Mantovani F. Gaggioli A. (2016). Transforming experience: the potential of augmented reality and virtual reality for enhancing personal and clinical change. *Front. Psychiatry* 7:164. 10.3389/fpsyt.2016.00164 27746747 PMC5043228

[B39] Riva G. Wiederhold B. K. Mantovani F. (2019). Neuroscience of virtual reality: from virtual exposure to embodied medicine. *Cyberpsychol. Behav. Soc. Netw.* 22 82–96. 10.1089/cyber.2017.29099.gri 30183347 PMC6354552

[B40] Slater M. Pérez Marcos D. Ehrsson H. Sanchez-Vives M. V. (2008). Towards a digital body: the virtual arm illusion. *Front. Hum. Neurosci.* 2:6. 10.3389/neuro.09.006.2008 18958207 PMC2572198

[B41] Schaverien J. (2000). “The triangular relationship and the aesthetic counter transference in analytical art psychotherapy,” in *The Changing Shape of Art Therapy: New Developments in Theory and Practice*, eds Gilroy A. McNeilly G. (London: Jessica Kingsley Publishers), 55–83.

[B42] Shalom J. G. Israeli H. Markovitzky O. Lipsitz J. D. (2015). Social anxiety and physiological arousal during computer mediated vs. face to face communication. *Comp. Hum. Behav.* 44 202–208. 10.1016/j.chb.2014.11.056

[B43] Shwartz H. (2000). *Dialogue With Forgotten Voices: Relational Perspective on Child Abuse Trauma and Treatment of Dissociative Disorder.* New York, NY: Basic Books.

[B44] Siano J. (2016). *Holy Junk: Lost, Found and Rejected Objects in Art Therapy.* Haifa: Emili Segol.

[B45] Yedidia T. Levine-Keini N. (2005). Making use of a family collage as a means for processing traumatic experiences working with Holocaust survivors. in *The Art of Ageing: Textualizing the Phase of Life*, eds Worsfold B. Kirkwood T. (Lleida: Universitat de Lleida), 165–176.

[B46] Winnicott D.W. (1971). *Playing and Reality.* New York, NY: Basic Books.

